# Mindfulness- and acceptance-based interventions for music performance anxiety: a three-level meta-analysis of therapeutic effects, mechanisms, and boundary conditions

**DOI:** 10.3389/fpsyg.2026.1851221

**Published:** 2026-06-11

**Authors:** Chengyu Zhang, Xin Shan, Huasen Zhang, Mengqi Zhang, Hao Zhou

**Affiliations:** 1 The Graduate School Arts and Culture, Sangmyung University, Seoul, Republic of Korea; 2 The Graduate School and Education, Sangmyung University, Seoul, Republic of Korea; 3 Art Education Center, Nanjing University of Finance and Economics, Nanjing, China

**Keywords:** acceptance interventions, attentional control, decentering, mindfulness, music performance anxiety, psychotherapy, therapeutic mechanisms

## Abstract

**Introduction:**

Music performance anxiety (MPA) can substantially impair musicians’ learning, performance, and psychological functioning. Although mindfulness- and acceptance-based interventions (MABIs) have shown promise for MPA, their pooled effects, boundary conditions, and mechanism-related commonalities remain insufficiently understood. Grounded in a dual-path explanatory framework emphasizing decentering and attentional reallocation, this study examined the effects of MABIs on anxiety-related outcomes and positive psychological functioning.

**Methods:**

A dual-track synthesis combining three-level meta-analysis and qualitative synthesis was conducted. Thirty-two independent studies (*N* = 1,983) were included in the qualitative synthesis, and 26 studies provided data suitable for quantitative meta-analysis. Three-level meta-analytic models were used to account for dependent effect sizes, and robust variance estimation was applied as a secondary sensitivity approach. Exploratory moderator and outcome-family analyses examined intervention type, intervention dose, and narrower outcome domains.

**Results:**

MABIs were associated with a moderate-to-large reduction in music performance-related anxiety outcomes (Hedges’ *g* = 0.732) and a small-to-moderate improvement in positive psychological functioning (*g* = 0.350). Exploratory analyses showed stronger effects for MPA-specific anxiety than for broader anxiety/stress responses, whereas no clear differential effects emerged within positive psychological functioning. Intervention type and dose did not significantly moderate the main effects. The qualitative synthesis identified four recurring therapeutic components: cognitive defusion, somatic anchoring, acceptance/values clarification, and self-compassion.

**Discussion:**

The findings suggest that MABIs may reduce anxiety and support adaptive psychological functioning among music performers. However, mechanism-related interpretations remain inferential because they are based on theoretical integration and qualitative evidence rather than direct mediation testing. Future research should use longitudinal designs, stronger control conditions, and multimodal physiological assessments to examine dose-response patterns and therapeutic pathways more directly.

**Systematic review registration:**

https://osf.io/7yr5s/overview, identifier osfregistrations-7yr5s-v1.

## 1 Introduction

Music performance anxiety (MPA) is a highly prevalent and functionally impairing form of state anxiety among musicians, substantially constraining both learning trajectories and professional development (Burin and Osório, 2017; Fernholz et al., 2019). In response to this challenge, traditional interventions, which have tended to focus either on regulating physiological arousal or on modifying anxiety-related cognitions, have often shown limited effectiveness (Kenny, 2005). In recent years, both clinical psychology and music education have increasingly shifted toward transdiagnostic intervention frameworks centered on psychological flexibility (Kaleńska-Rodzaj, 2021; Kinney et al., 2025). As a representative example of this framework, mindfulness- and acceptance-based interventions (MABIs) comprise a class of psychological interventions that emphasize nonjudgmental awareness of present-moment experience, that is, mindfulness (Kabat-Zinn, 2003), and an open willingness to engage with internal discomfort, that is, acceptance (Hayes et al., 2006). Rather than seeking to forcibly eliminate symptoms or alter the content of thoughts, it aims to reshape the individual’s relationship with their internal experiences, enabling them to pursue meaningful goals even in the presence of anxiety; this approach demonstrates potential practical value within a musical context (Hayes et al., 2006; Juncos et al., 2017).

As MABIs have been increasingly adopted in music settings, their surface forms have become highly diverse. However, the existing literature has largely evaluated individual intervention approaches in isolation, and few have examined whether these superficially distinct interventions involve format-specific elements, shared therapeutic components, or both. Recent theoretical work suggests that the effects of MABIs may be partly related to common processes such as decentering and mindful awareness (Bos et al., 2024). This focus is also consistent with recent science-mapping evidence showing that interoceptive awareness and mindfulness research has increasingly moved toward stress-reduction interventions, clinical applications, and physiological-measurement themes (Nguyen and Tran, 2026). Therefore, recurring therapeutic components and their plausible links with MPA-related processes warrant further investigation.

In addition, the effects of MABIs may be constrained by important boundary conditions. First, most existing evaluations have focused on reducing performance anxiety. However, the dual-continua model suggests that reduced distress does not necessarily imply psychological flourishing (Keyes, 2002). Therefore, this review also examined positive psychological functioning as an umbrella outcome domain, including adaptive psychological capacities and positive experiential states that performers may develop under pressure. This broader scope is consistent with recent science-mapping work showing that mindfulness research is increasingly connected with psychological wellbeing and broader health-related priorities (Nguyen and Tran, 2025b). This broader outcome domain helps clarify the potential boundaries of MABI effects. Second, it remains unclear whether the effects of MABIs follow a linear dose–response pattern; to date, the evidence remains inconclusive (Strohmaier, 2020).

Previous reviews and meta-analyses have provided important evidence on interventions for music performance anxiety and related forms of performance anxiety. Burin and Osório (2016) and Kinney et al. (2025) systematically summarized a broad range of interventions for MPA, including CBT, biofeedback, yoga, meditation, music therapy, and the Alexander Technique, while also noting persistent methodological limitations such as small samples and weak study designs. Bakhtiari et al. (2025) further reviewed recent coping strategies for MPA, including ACT, CBT, mindfulness, and yoga; however, because of heterogeneity in study designs and outcome measures, their review used narrative synthesis rather than meta-analysis and therefore did not quantitatively estimate pooled intervention effects or moderator effects. Broader meta-analytic evidence has examined psychological interventions for performance anxiety among performing artists and athletes, but this wider scope makes it difficult to isolate the specific evidence for MPA and for mindfulness- and acceptance-based interventions in music contexts (Niering et al., 2023).

Building on this evidence base, the present study extends prior work in several ways. Specifically, it focuses on mindfulness- and acceptance-based interventions for MPA, uses three-level meta-analysis to estimate their overall effects on anxiety-related outcomes and positive psychological functioning, and further examines potential boundary conditions such as intervention type and intervention dose. At the same time, the study incorporates qualitative synthesis to identify recurring therapeutic components across different intervention formats and to integrate mechanism-related evidence. Through this dual-track design, the present review supplements the quantitative effect evidence that remains limited in previous narrative reviews and further examines the shared therapeutic components through which MABIs may operate in the context of MPA. Based on this design, the present study addresses the following core research questions:

*RQ1*: Can MABIs significantly reduce music performance-related anxiety among music performers as the primary outcome?

*RQ2*: Can MABIs significantly enhance performers’ broad adaptive positive psychological functioning, operationalized as an umbrella domain of positive psychological functioning?

*RQ3*: To what extent do intervention characteristics, including intervention type and intervention dose, moderate the effect size of MABIs on anxiety?

*RQ4*: What core therapeutic components are embedded in existing intervention models, and how do these components exert their effects by targeting specific psychopathological processes?

## 2 Explanatory framework

### 2.1 Reperceiving model

The reperceiving model proposed by Shapiro et al. (2006) posits that the key mechanism underlying MABIs lies in a metacognitive shift in how individuals relate to their internal experiences, a process that has subsequently been widely interpreted as closely related to decentering. In the context of music performance, MABIs do not aim to forcibly eliminate performers’ expectations of mistakes or physiological arousal. Rather, they train performers to approach these experiences with a more observant and defused stance, that is, to regard tension as a temporary internal psychological event rather than as objective evidence of impending failure. Empirical evidence suggests that this fundamental shift in one’s relationship to internal experience not only precedes but also significantly predicts improvements in anxiety symptoms (Hayes-Skelton et al., 2015), but may also further enhance self-regulation and cognitive-behavioral flexibility, thereby broadly promoting gains in positive psychological functioning (Carmody et al., 2009).

### 2.2 Attentional control theory (ACT)

ACT proposed by Eysenck et al. (2007) focuses on the competitive dynamics between the goal-directed system and the stimulus-driven system under high-pressure conditions. Anxiety particularly impairs the inhibitory and shifting functions of the executive system, thereby increasing attentional bias toward threat-related information. From this perspective, the intervention logic of MABIs lies in repeated attentional re-anchoring practice, such as focusing on the breath or bodily kinesthetic feedback, which helps performers bring attention back in a nonjudgmental manner once mind wandering is detected (Bishop et al., 2004; Jha et al., 2007). This process enables performers to maintain task-related focus and a sense of control over the musical task even under pressure, thereby providing a cognitive foundation for symptom reduction and the development of adaptive functioning.

### 2.3 An integrated dual-path framework and research hypotheses

**FIGURE 1:**
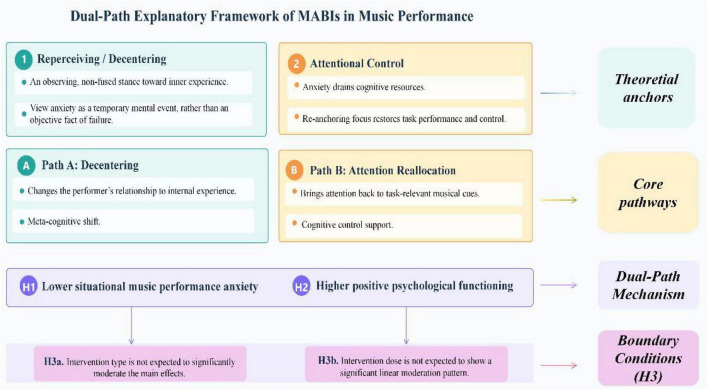
Dual-path explanatory framework of MABIs in music performance.

Building on the foregoing theories, the present study proposes a dual-path explanatory framework to integrate the mechanisms through which MABIs operate in music performance contexts: namely, decentering at the metacognitive level (i.e., changing one’s relationship to experience) and attentional resource reallocation at the cognitive level (i.e., maintaining task engagement). These two pathways may jointly inform how MABIs are expected to influence anxiety-related and adaptive-functioning outcomes in music performance contexts. Based on this framework (see Figure 1), the present study advances the following hypotheses:

*H1:* MABIs significantly reduce MPA in music performance-related anxiety situations.

*H2:* MABIs significantly enhance performers’ positive psychological functioning.

Furthermore, to examine potential boundary conditions in practical applications, this study tested whether intervention type and dose were associated with variation in effect sizes. Because different MABI formats may share therapeutic components while differing in delivery procedures, intervention type was examined as an exploratory moderator. Accordingly:

*H3a:* Intervention type is associated with variation in the effect of MABIs on anxiety outcomes.

In addition, it remains unclear whether intervention dose is linearly associated with outcome change. Given the broad dose categories available in the current literature, intervention dose was also examined as an exploratory moderator.

*H3b:* Intervention dose is associated with variation in the effect of MABIs on anxiety outcomes.

## 3 Materials and methods

### 3.1 Literature search and study selection

This systematic review and meta-analysis was conducted and reported in strict accordance with the Preferred Reporting Items for Systematic Reviews and Meta-Analyses 2020 guidelines (Page et al., 2021), and was preregistered on the Open Science Framework (OSF). In February 2026, a systematic search was conducted across four major databases: Web of Science, Scopus, APA PsycINFO/PsycArticles, and ERIC. The search strategy combined controlled vocabulary terms and free-text terms and used Boolean operators to cross-search three core concept clusters: (1) study context, (2) intervention, and (3) outcome variables. Search query: music AND (Perform* OR Stage OR Recital OR Concert OR Audition OR Rehearsal) AND (Mindful* OR Meditat* OR Yoga OR MBSR OR MBCT OR ACT OR “Acceptance and commitment” OR “Body scan” OR FFMQ OR MAAS) AND (Anxi* OR fright OR Distress OR Tension OR MPA OR “Performance anxiety” OR “Wellbeing” OR Wellbeing OR “Quality of life” OR Wellness OR “Mental health” OR Satisfaction OR Happiness OR “Positive affect” OR Flourishing OR Thriving OR Eudaimoni* OR “Personal growth” OR SWB OR PWB OR “Self-acceptance” OR “Meaning in life” OR Purpose OR Flow OR “Optimal experience” OR “Peak performance” OR Resilien* OR Engag*).

**FIGURE 2:**
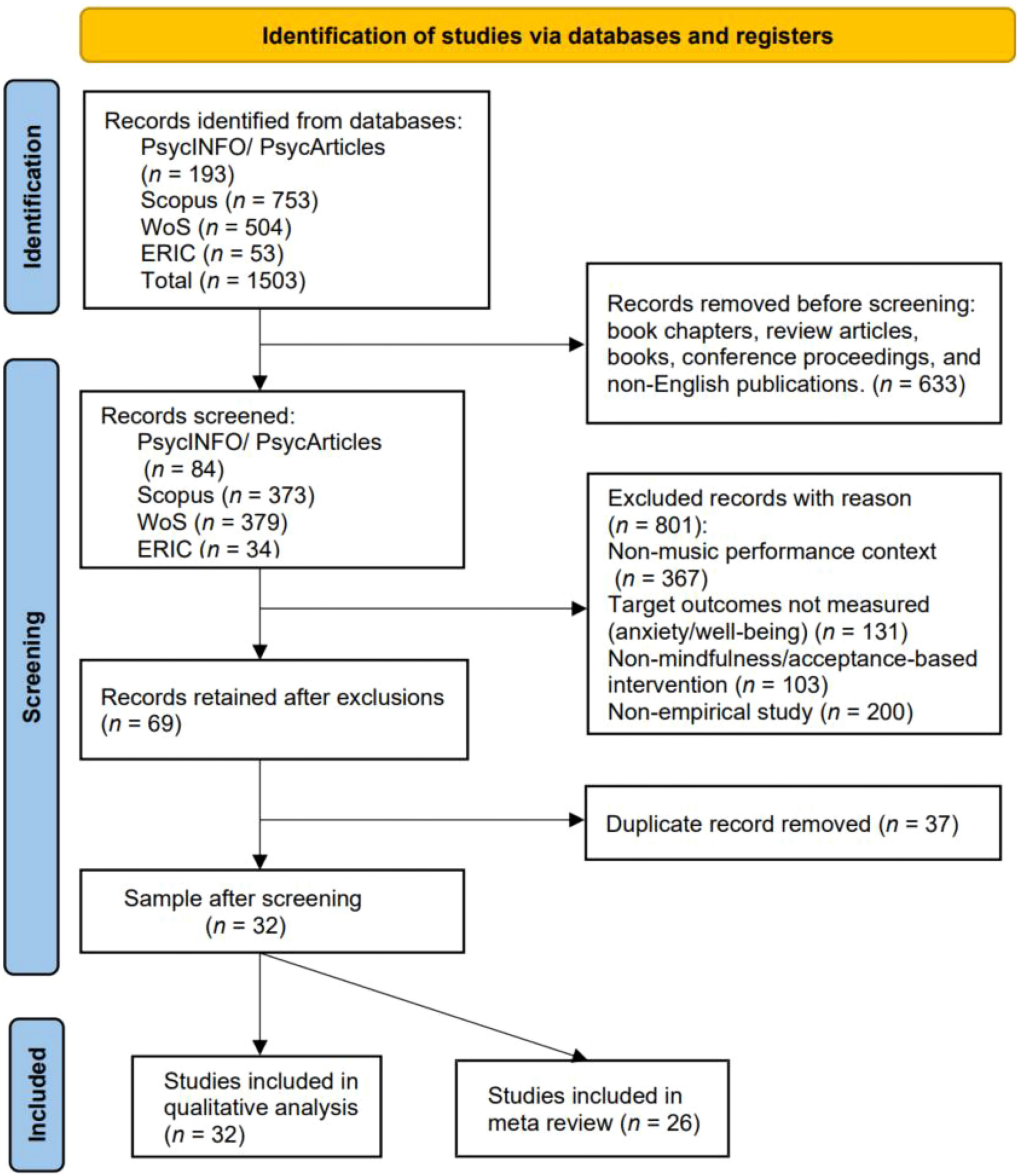
PRISMA flow diagram of study selection.

The detailed screening process is presented in Figure 2, the PRISMA flow diagram. The full screening record and the final list of included studies have been openly shared on the OSF platform.

### 3.2 Eligibility criteria for the analytic streams

This study adopted a dual-track evidence synthesis design. During title/abstract screening and full-text screening, all records first followed the same common topic-level eligibility criteria: studies had to focus on mindfulness-, acceptance-, meditation-, yoga-, or compassion-based interventions in music performance-related contexts and report outcomes related to music performance anxiety, anxiety/stress responses, or adaptive psychological functioning. The 32 empirical studies that met these common topic-level criteria were all included in the mechanism-oriented qualitative synthesis to address RQ4. Specifically, all studies coded as S1–S32 in Supplementary Table 1 were included in the qualitative synthesis, whereas S1–S26 were further included in the quantitative meta-analysis because they provided data suitable for calculating standardized pre-post effect sizes. Therefore, the dual-track design did not classify the literature into two mutually exclusive categories. Rather, the 32 empirical studies constituted the overall evidence base, from which the studies meeting the statistical requirements were extracted to form the quantitative meta-analytic subset.

The quantitative meta-analysis adopted eligibility criteria based on the PICOS framework (see Table 1). Considering that music performance intervention research is often constrained by authentic educational settings, specialized sample sizes, curriculum arrangements, and ethical feasibility, the number of rigorous randomized controlled trials remains limited. Previous reviews have also noted that high-quality intervention evidence in this field is still relatively insufficient (Kinney et al., 2025). Therefore, this study did not require random allocation or an independent control group as a necessary condition for inclusion in the quantitative meta-analysis. Instead, the core criterion was whether a study provided data from which pre–post intervention effect sizes could be calculated. In other words, empirical intervention studies were eligible for inclusion in the quantitative three-level meta-analysis as long as they reported pre- and post-intervention measurement data, including means, standard deviations, sample sizes, or other statistical information convertible into standardized effect sizes. At the same time, differences in study design were not ignored; rather, they were addressed through risk-of-bias assessment, study-design covariate analysis, and cautious interpretation of the findings, in order to avoid treating evidence from different study designs as equivalent in evidential strength.

**TABLE 1:** Eligibility criteria for the quantitative meta-analysis based on the PICOS framework.

Dimension	Inclusion criteria	Exclusion criteria
Population	Musicians, music students, music learners, performers, or individuals situated in music performance-related contexts.	Non-music performers; participants unrelated to music performance or music learning contexts.
Intervention	Mindfulness, meditation, yoga, acceptance and commitment therapy, compassion training, or multicomponent interventions containing the above core elements.	Interventions not grounded in mindfulness- or acceptance-based approaches; general relaxation training without mindfulness, acceptance, or awareness-based components.
Comparator	Randomized control groups, nonrandomized control groups, active control groups, waitlist control groups, treatment-as-usual or regular instruction control groups, or single-group pretest-posttest designs.	Studies without any pre-post intervention comparison information.
Outcomes	Outcomes related to anxiety or adaptive psychological functioning, including music performance anxiety, state anxiety, general anxiety, stress responses, emotion regulation, mindfulness, psychological flexibility, self-efficacy, wellbeing, resilience, flow, and positive affect.	Studies that did not report any outcome related to anxiety, stress, or adaptive psychological functioning.
Study design	Empirical intervention studies, including RCTs, nonrandomized controlled studies, quasi-experimental studies, single-group pretest-posttest studies, and other intervention designs from which pre-post data could be extracted.	Intervention studies that did not provide pre-post data.
Data requirement	Studies reporting pre- and post-intervention means, standard deviations, and sample sizes, or other statistical information that could be converted into effect sizes.	Studies that did not provide sufficient statistical information and for which effect sizes could not be calculated or obtained by contacting the authors.
Language and publication type	Peer-reviewed journal articles published in English.	Full text unavailable; non-English publications; retracted articles.

### 3.3 Outcome definition

For anxiety-related outcomes, this study treated MPA-specific anxiety as the core outcome family, primarily including measures that directly assessed music performance anxiety or anxiety in performance-related contexts. Common instruments included the K-MPAI, PAQ, PAI, and MPAI-A. When state anxiety, trait anxiety, generalized anxiety, perceived stress, physiological stress responses, or emotion dysregulation were explicitly situated within the context of music performance pressure or intervention effects, they were coded as broader anxiety-related responses. Relevant measures included the STAI, GAD-7, DASS-21, POMS, physiological indicators, and emotion dysregulation measures.

For positive psychological functioning, this outcome domain was used to capture the broader adaptive psychological functioning that MABIs may promote in music performance stress contexts. The theoretical rationale is that MABIs do not merely aim to reduce anxiety symptoms. Rather, through mindful awareness, acceptance, decentering, somatic anchoring, cognitive defusion, and values-guided action, they may help performers regulate internal experiences, sustain task engagement, restore psychological resources, and develop more adaptive psychological functioning under high-pressure performance conditions (Hayes et al., 2006; Hölzel et al., 2011; Johannsen et al., 2022). Consistent with the logic of prior meta-analyses of psychological interventions, these diverse constructs were conceptually integrated into the broader domain of positive psychological functioning in order to capture improvements and transformations in adaptive cognitive, emotional, and experiential processes more comprehensively (Fredrickson, 2001; Kashdan and Rottenberg, 2010; Ryff, 1989).

To reduce the interpretive risk associated with conceptual heterogeneity, positive psychological functioning was further divided into two narrower outcome families. The first family comprised proximal adaptive processes, referring mainly to outcomes that more directly reflected the core training mechanisms of MABIs, such as mindfulness skills, present-moment awareness, psychological flexibility, and related processes, including acceptance, cognitive defusion, emotion regulation, and self-compassion. Relevant measures mainly included the FFMQ, MAAS, Freiburg Mindfulness Inventory, AAQ-II, MPFI, and SCS. The second family comprised distal performance and wellbeing resources, referring to positive psychological resources, performance confidence, and positive experiential states that may further emerge following intervention. This family mainly included self-efficacy, performance confidence, psychological wellbeing, resilience, positive affect, and flow. Relevant measures included performance confidence or self-efficacy indicators, SPWS, WEMWBS, Ryff PWB/RPWS, MHC, DFS-2, and other indicators related to wellbeing, positive affect, or flow experience.

### 3.4 Data analysis

To ensure systematicity and internal consistency in quality appraisal, all studies were evaluated using the JBI critical appraisal tools (JBI, 2023). In the quantitative synthesis, to address RQ1 and RQ2, standardized mean differences (SMDs) were extracted and pooled, with Hedges’ g used to correct for small-sample bias. Because multiple primary studies reported several related effect sizes within the same sample (e.g., both cognitive anxiety and somatic anxiety), the data exhibited nested dependence. Ignoring this hierarchical structure could underestimate standard errors and artificially inflate statistical significance. Therefore, this study employed the three-level meta-analysis model proposed by Cheung (2014), which decomposes variance into three levels: Level 1, sampling variance; Level 2, within-study variance across multiple effect sizes from the same study; and Level 3, between-study variance across independent studies. All models were estimated in the metafor package in R (Viechtbauer, 2010).

As a secondary robustness check for effect-size dependence, robust variance estimation (RVE) with small-sample correction was also applied (Tipton, 2015). Under the correlated-effects model, the within-study correlation among outcome measures was set at ρ = 0.80, and additional sensitivity analyses were conducted across a plausible range of ρ values to ensure the robustness of the findings. In addition, leave-one-out influence analyses were conducted for the two main umbrella outcome domains to examine whether the pooled estimates were disproportionately driven by any single study. This approach follows established influence-diagnostic procedures in meta-analysis, in which each study is omitted in turn and the stability of the pooled estimate is inspected (Viechtbauer and Cheung, 2010).

To clarify the interpretive boundaries of the two umbrella domains, exploratory outcome-family analyses were further conducted. For anxiety-related outcomes, effect sizes were classified into MPA-specific anxiety and broader anxiety/stress-related responses. For positive psychological functioning, effect sizes were classified into proximal adaptive processes and distal performance- and wellbeing-related resources. These analyses were intended to examine whether the overall pooled effects were concentrated in specific narrower outcome families, rather than to provide confirmatory tests of distinct mechanisms.

Finally, for the two main umbrella outcome domains, potential publication bias and small-sample effects were assessed using visual inspection of the funnel plot together with Egger’s linear regression test (Egger et al., 1997).

## 4 Results

### 4.1 Risk of bias, funding, and conflict of interest

This study used JBI critical appraisal tools matched to study design to assess the risk of bias in the 32 included studies. For mixed-methods studies, the corresponding JBI tool was selected according to the primary study design or the component that made the greatest contribution to the main evidence stream of this review, and a single study-level overall appraisal was conducted. For each study, each JBI item was first rated as “Yes,” “No,” “Unclear,” or “Not applicable.” In the overall risk-of-bias judgment, “Not applicable” items were not included in the denominator when calculating the proportion of “Yes” ratings; “No” indicated a clear methodological limitation for that item; and “Unclear” indicated that the reported information was insufficient to determine whether the item was satisfied. The overall risk-of-bias level was determined based on the proportion of “Yes” ratings among applicable items and the severity of deficiencies in key items. The specific criteria are shown in Table 2.

**TABLE 2:** Criteria for overall risk-of-bias judgments based on JBI appraisal.

Proportion of “Yes” ratings	Overall risk of bias
≥ 80% “Yes” ratings, with no major concerns in key domains	Low risk
70–79% “Yes” ratings, or only a small number of “Unclear” ratings	Low-to-moderate risk
60–69% “Yes” ratings, or one clear concern in a key domain	Moderate risk
45–59% “Yes” ratings, or two or more clear concerns in key domains	Moderate-to-high risk
< 45% “Yes” ratings, or serious flaws in key design domains	High risk

Across the 32 included studies, 1 study was rated as having low risk of bias, 12 studies as low-to-moderate risk of bias, 8 studies as moderate risk of bias, 10 studies as moderate-to-high risk of bias, and 1 study as high risk of bias (see Table 3). The main risk-of-bias concerns in the existing studies were concentrated in quantitative intervention studies. Specifically, the main limitations included the absence of an independent control group in some studies, making it difficult to rule out time effects or natural recovery effects (e.g., S1 and S3); the use of nonrandomized allocation in some studies, resulting in insufficient baseline comparability between groups (e.g., S9 and S15); and small sample sizes, insufficient reporting of follow-up procedures, or insufficient reporting of outcome-measure reliability in several studies (e.g., S5 and S14).

**TABLE 3:** Summary of JBI risk-of-bias ratings by study design.

Study design	JBI tool	*n*	Low	Low–moderate	Moderate	Moderate–high	High	Main concern
RCT/RCT component	JBI critical appraisal checklist for RCTs	5	0	0	5	0	0	Blinding of participants and intervention providers was generally not feasible; allocation concealment, assessor blinding, follow-up completeness, and intention-to-treat analysis were sometimes unclear.
Quasi-experimental, CCT, and single-arm pre-post component	JBI checklist for Quasi-experimental studies	19	0	6	3	9	1	Common limitations included absence of control groups in single-arm studies, unclear group comparability, limited repeated pre/post measurements, incomplete follow-up reporting, and unclear reliability of some outcome measures.
Analytical cross-sectional study	JBI analytical cross-sectional checklist	1	1	0	0	0	0	Reporting was generally complete, with clearly defined sample criteria, valid measurement, and appropriate statistical analysis.
Qualitative study/qualitative component	JBI checklist for qualitative research	3	0	2	0	1	0	Main concerns were limited reporting of philosophical positioning, researcher reflexivity, and the researcher–participant relationship.
Single-subject/case-report/case-study evidence	JBI case report checklist	4	0	4	0	0	0	Demographic, intervention, and post-intervention descriptions were generally clear, but adverse events were not reported and causal inference remained limited because of the case-based design.

Overall, the current evidence base is more appropriately understood as moderate-strength evidence rather than definitive causal evidence. Considering the limited number of music performance intervention studies and the constraints imposed by authentic music education and performance-training ecologies, this review did not use risk-of-bias ratings as a mechanical exclusion criterion. In addition, this study conducted leave-one-out influence analyses to examine whether any single study exerted a disproportionate influence on the pooled effects.

With regard to funding and conflict of interest, all included studies were supported by purely academic grants from governments, universities, or nonprofit institutions, and no evidence was found of systematic bias associated with commercial sponsorship. In the few studies involving potential conflicts of interest (e.g., authors serving as intervention developers: S9 and S32; or authors employed by yoga organizations: S24 and S25), these risks were addressed through transparent disclosure or independent third-party data handling. Overall, the current empirical foundation is more appropriately characterized as moderate-strength evidence rather than a highly conclusive causal evidence chain.

### 4.2 Descriptive results

**FIGURE 3:**
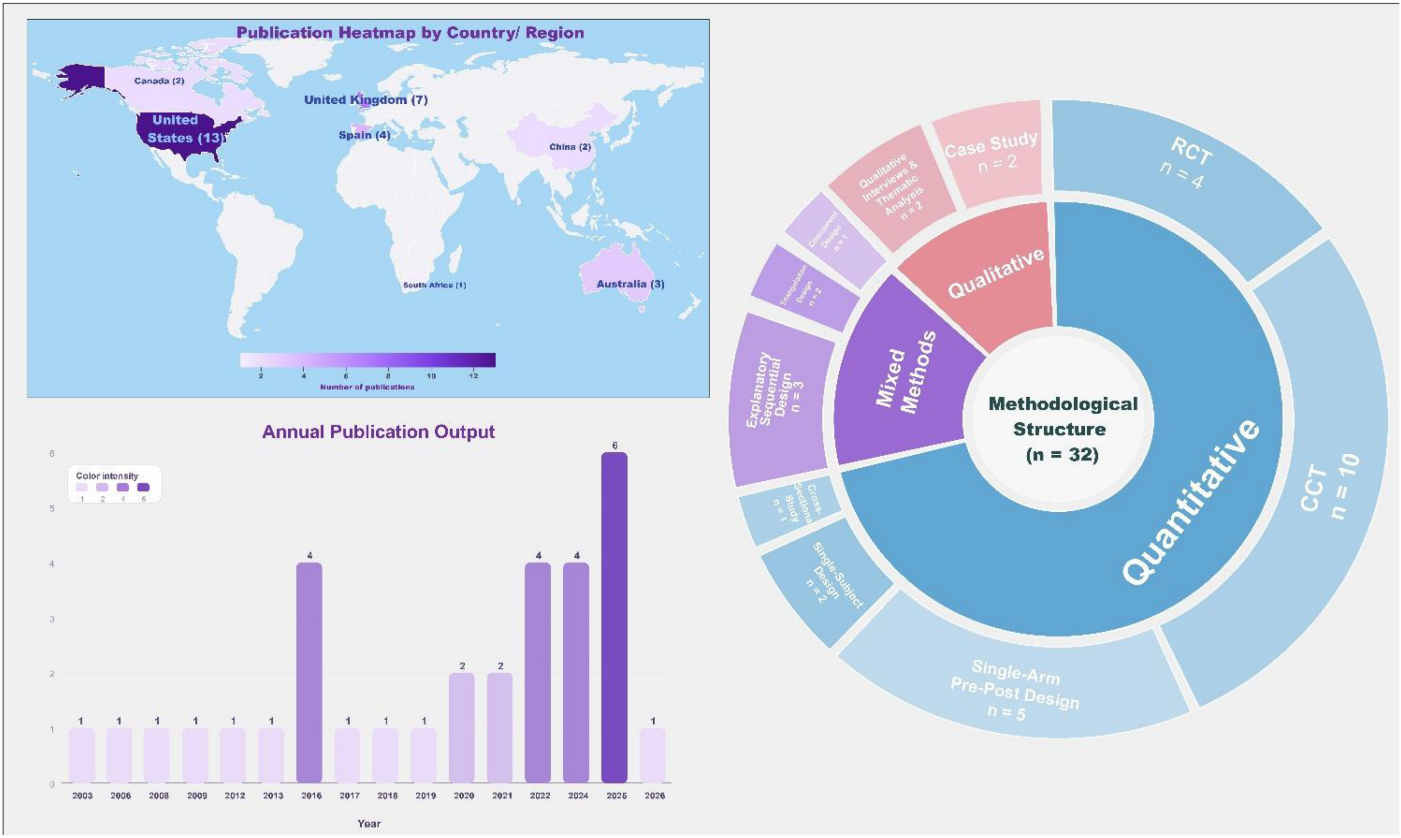
Descriptive characteristics of the included studies.

A total of 32 independent studies met the inclusion criteria, with publication years ranging from 2003 to 2026 (see Figure 3) (detailed characteristics are shown in Supplementary Table 1). Notably, more than half of the studies (approximately 62.5%) were published in or after 2020, indicating a marked increase in research interest in MABIs in the domain of music performance.

Geographically, the included studies reflected broad international coverage. The United States (*n* = 11) and the United Kingdom (*n* = 6) were the leading contributors, followed by Spain (*n* = 3), Australia (*n* = 3), China (*n* = 2), Canada (*n* = 2), and several other countries, including Germany, South Africa, and New Zealand. This distribution suggests substantial cross-cultural interest in this topic across the fields of music education and psychology.

Across all studies, the review involved approximately 1,983 participants, with sample sizes ranging from *N* = 1 to *N* = 498. The samples consisted mainly of conservatory and university music students aged 18-30, with women generally overrepresented, but also extended to adolescent learners aged 13-17, middle-aged and older adults, and mature professional musicians.

In addition, the included literature showed a methodologically diverse pattern, although quantitative designs accounted for the largest proportion of the evidence base. Among the 32 included studies, 22 used quantitative designs, including randomized controlled trials (RCTs, *n* = 4), non-randomized controlled or quasi-experimental designs (CCTs, *n* = 10), single-arm pre-post designs (*n* = 5), single-subject designs (*n* = 2), and a cross-sectional survey design (*n* = 1). In addition, six studies used mixed-methods designs, and four studies used qualitative designs.

The mixed-methods studies mainly comprised three design types. Three studies used explanatory sequential designs, typically combining quantitative pre-post assessment with subsequent qualitative interviews or subjective feedback to further interpret participants’ intervention experiences and perceived processes of change. Two studies combined RCT designs with qualitative diary data and followed a parallel triangulation approach. One study integrated a cross-sectional questionnaire survey with qualitative analysis of open-ended responses. The qualitative studies included qualitative interview studies (*n* = 2) and case-study designs (*n* = 2). Overall, the current evidence base remains primarily quantitative, but the mixed-methods and qualitative studies provide complementary contextual information for understanding participants’ intervention experiences, subjective changes, and possible processes of change.

### 4.3 Overall intervention effects and three-level heterogeneity decomposition

#### 4.3.1 Robustness and publication-bias diagnostics

To examine whether the pooled effects were disproportionately influenced by any single study, leave-one-out sensitivity analyses were conducted separately for the two main umbrella outcome domains, namely music performance-related anxiety outcomes and positive psychological functioning outcomes (see Table 4). For anxiety-related outcomes, omitting each study in turn did not change the direction or statistical significance of the pooled effect; the largest shift occurred after omitting Wang (2025), with a signed change of Δ = −0.114. For positive psychological functioning outcomes, the pooled effect likewise remained positive and statistically significant after each study was removed in turn; the largest shift occurred after omitting Lee et al. (2019), with a signed change of Δ = −0.079. Overall, the leave-one-out analyses indicated that the pooled effects for both main outcomes were not driven by any single study.

**TABLE 4:** Robustness and publication-bias diagnostics for the main outcome domains.

Outcome domain	Full model estimate	Range of leave-one-out estimates	Largest shift after omitting	Direction/ significance changed?	Effect sizes used	Egger coefficient	*t*	*p*	Egger test conclusion
Anxiety-related outcomes	*g* = 0.732, 95% CI [0.514, 0.965]	*g* = 0.618–0.787	Wang, 2025; Δ = −0.114	No	69	−0.566	−0.42	0.681	Non-significant
Positive psychological functioning	*g* = 0.350, 95% CI [0.250, 0.450]	*g* = 0.271–0.402	Lee et al., 2019; Δ = −0.079	No	139	2.527	0.82	0.423	Non-significant

**FIGURE 4:**
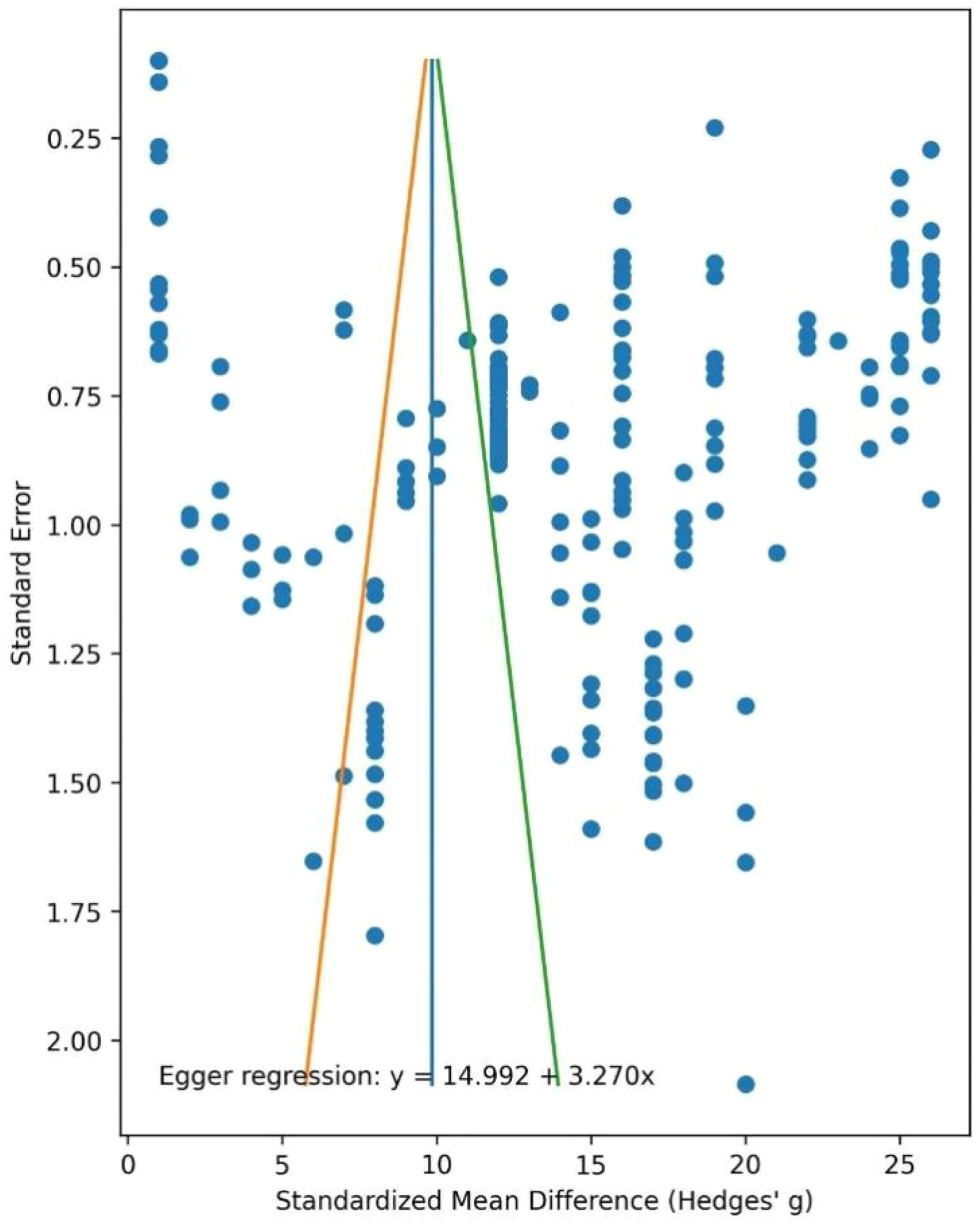
Funnel plot for publication bias assessment.

To further assess potential small-study effects and publication bias, publication-bias diagnostics were conducted using funnel plots and Egger’s linear regression tests. The funnel plots shown in Figure 4 present the overall distributions at the level of the two main umbrella outcome domains. Because several studies contributed multiple dependent effect sizes, Egger’s tests were conducted using study-clustered robust standard errors. Overall, the funnel plots showed some fluctuation in the distribution of small-sample studies, but no typical pattern of systematic asymmetry was observed. Egger’s tests were then conducted separately for the two main outcome domains. For anxiety-related outcomes, the test included 69 effect sizes from 18 studies; the Egger regression coefficient was −0.566, *t* = −0.42, *p* = 0.681, indicating no statistically significant result. For positive psychological functioning outcomes, the test included 139 effect sizes from 17 studies; the Egger regression coefficient was 2.527, *t* = 0.82, *p* = 0.423, which was also not statistically significant. However, these diagnostic results should be understood as supplementary robustness evidence rather than definitive evidence ruling out risk of bias.

#### 4.3.2 Effects of MABIs on music performance-related anxiety outcomes

**FIGURE 5:**
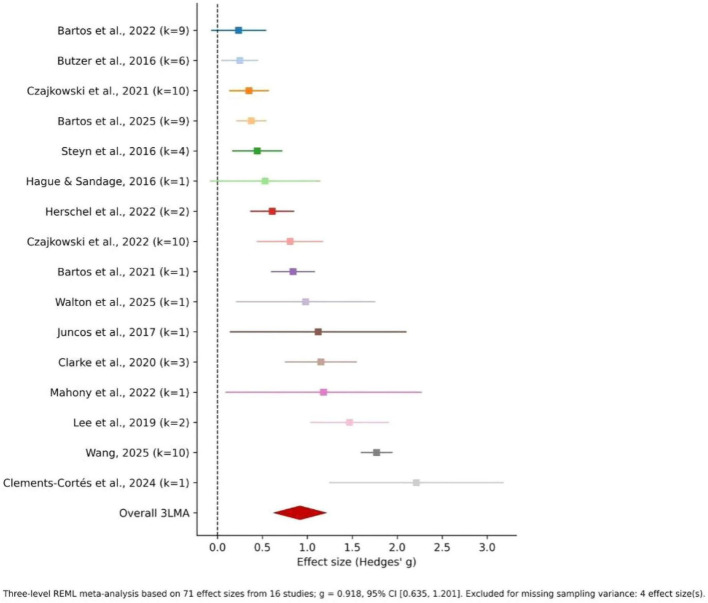
Main effect of MABIs on context-specific state anxiety.

The main three-level meta-analytic model showed that MABIs produced a statistically significant moderate-to-large overall effect in reducing music performance-related anxiety outcomes [*g* = 0.732, SE = 0.115, 95% CI (0.514, 0.965), *p* < 0.001] (see Figure 5). However, variance decomposition (total variance = 0.0479) revealed that the majority of this heterogeneity was attributable to between-study differences (Level 3 variance = 0.0383) rather than within-study variations (Level 2 variance = 0.0096).

To address this observed heterogeneity and the potential conceptual broadness of the overall domain, exploratory sensitivity analyses were conducted on two narrower outcome families. Significant effects emerged for both: MPA-specific anxiety yielded a relatively large effect [*g* = 0.982, SE = 0.312, 95% CI (0.372, 1.593), *p* = 0.002; 48 effect sizes from 15 studies], whereas broader anxiety/stress responses showed a smaller effect [*g* = 0.345, SE = 0.082, 95% CI (0.185, 0.506), *p* < 0.001; 21 effect sizes from 6 studies]. Exploratory meta-regression further indicated that the effect was significantly stronger for MPA-specific anxiety than for broader anxiety/stress responses (*b* = 0.637, SE = 0.322, *p* = 0.048).

#### 4.3.3 Effect of MABIs on the umbrella domain of positive psychological functioning

**FIGURE 6:**
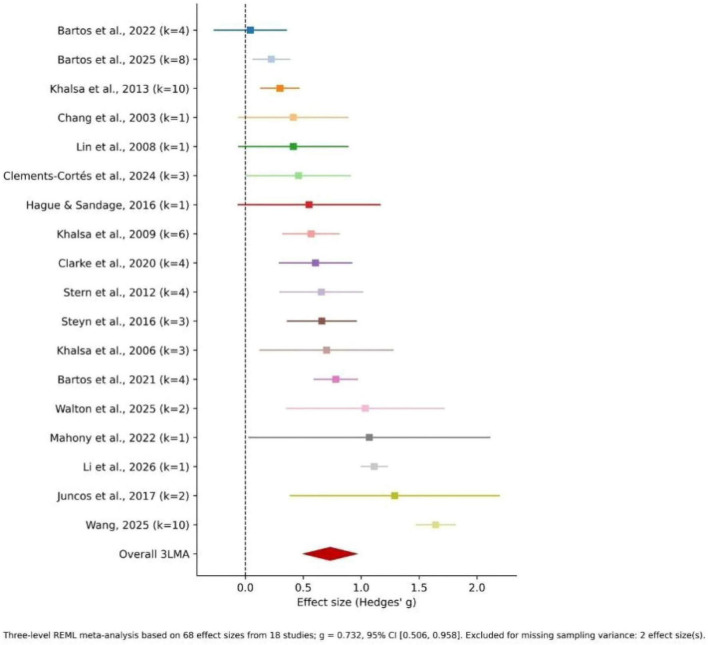
Main effect of MABIs on positive psychological functioning.

For the umbrella domain of positive psychological functioning, the analysis included 17 independent studies covering both proximal adaptive processes and distal performance- and wellbeing-related resources. The pooled effect size indicated that MABIs produced a significant positive effect on this broad adaptive-functioning outcome domain among music performers [*g* = 0.35, SE = 0.05, 95% CI (0.250, 0.450)] (see Figure 6). Within the three-level meta-analytic framework, this outcome also showed significant heterogeneity (*I*^2^ = 65%, *p* < 0.05). The variance decomposition indicated variability both between studies (Level 3 variance = 0.15) and among multiple effect sizes within the same study (Level 2 variance = 0.03).

To further examine the internal structure of the positive psychological functioning umbrella domain, an exploratory outcome-family meta-regression was conducted by classifying outcomes into proximal adaptive processes and distal performance and wellbeing resources. The pooled effect for proximal adaptive processes was statistically significant, *g* = 0.883, SE = 0.287, 95% CI [0.319, 1.446], *p* = 0.002, based on 78 modeled effect sizes. The pooled effect for distal performance and wellbeing resources was also statistically significant, *g* = 0.594, SE = 0.117, 95% CI [0.364, 0.824], *p* < 0.001, based on 61 effect sizes. However, the moderator effect of outcome family was not statistically significant, *b* = 0.288, SE = 0.279, *p* = 0.302. Accordingly, although both narrower outcome families showed positive effects, the present evidence does not support a clear differential effect between them.

#### 4.3.4 Robust variance estimation and moderator analysis

To identify potential sources of heterogeneity and address RQ3, the present study conducted a two-step analysis using RVE and multilevel meta-regression.

**FIGURE 7:**
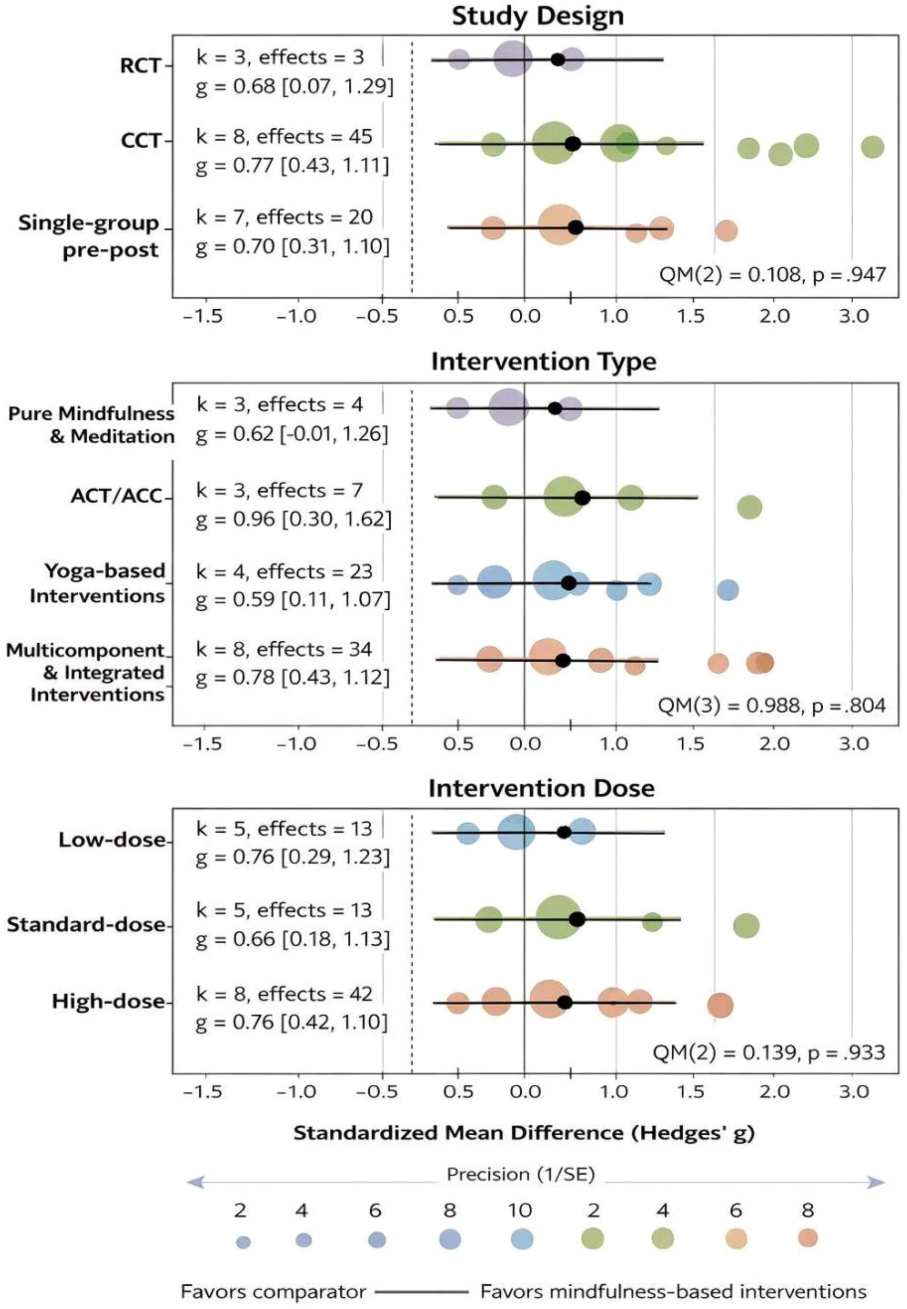
Moderator analyses by study design, intervention type, and intervention dose.

First, to further verify the reliability of the three-level model in handling data dependence, an RVE sensitivity analysis was conducted with ρ = 0.80. The results were highly consistent with those of the main model (see Supplementary Table 2 and Figure 7). In addition, study design was entered into the meta-regression as a methodological covariate, with studies categorized as RCTs, CCTs, and single-group pretest-posttest designs. The results showed that study design did not significantly moderate the intervention effect, QM(2) = 0.108, *p* = 0.947.

Second, after controlling for methodological variation, the study proceeded to test the theoretical boundary conditions of the dual-path explanatory framework.

MABIs were classified into four categories: Pure mindfulness and meditation, ACT/ACC, yoga-based interventions, and multicomponent and integrated interventions. The results indicated that neither the specific intervention tradition nor the mode of combination significantly moderated the intervention effect, QM(3) = 0.988, *p* = 0.804.

Total intervention dose was classified into three levels: low dose (1–7 sessions), standard dose (8–12 sessions), and high dose (13 or more sessions, or interventions spanning an entire semester/academic year). The results likewise showed no significant moderating effect of intervention dose, QM(2) = 0.139, *p* = 0.933.

### 4.4 Core therapeutic components and targeted psychopathological processes

**FIGURE 8:**
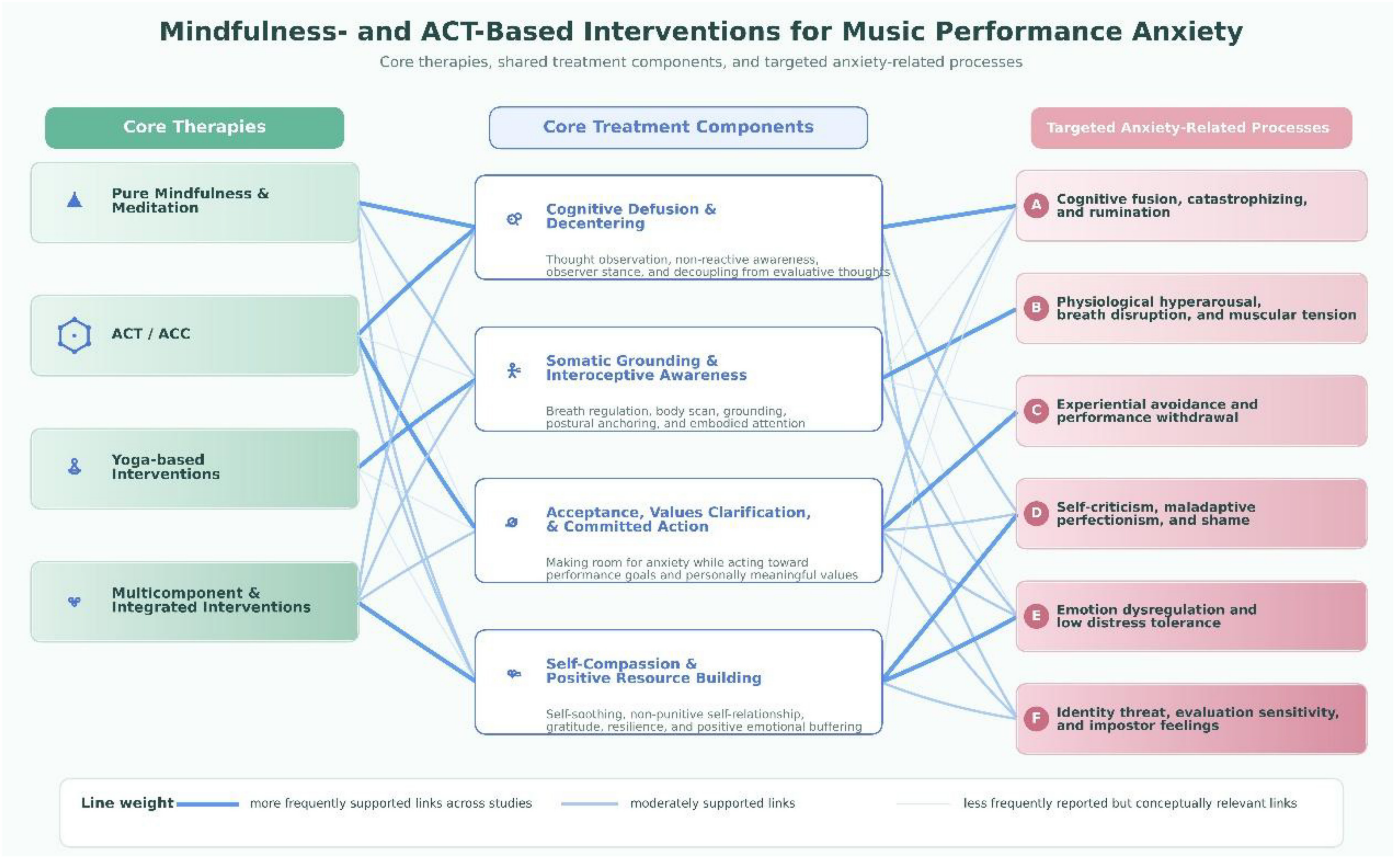
Core therapeutic components of MABIs for MPA.

Mindfulness- and acceptance-based interventions for MPA, particularly those grounded in ACT, do not primarily aim to eliminate surface-level symptoms of tension. Rather, they seek to enhance overall psychological flexibility by reshaping individuals’ psychological relationship with anxiety. Across the 32 empirical studies reviewed, these interventions appeared to involve four core therapeutic components that target the cognitive, physiological, behavioral, and self-related psychopathological processes underlying MPA (see Figure 8).

First, cognitive defusion and decentering represent one of the core therapeutic components, mainly targeting cognitive fusion, catastrophic rumination, and attentional bias toward threat. Under performance pressure or after unexpected mistakes, musicians often become trapped in intense self-criticism and repetitive replay of errors. The reviewed studies suggest that mindfulness practices such as observing thoughts, nonreactive awareness, and training in an observer stance may help individuals experience such thoughts as transient mental events rather than commands that must be followed (e.g., S9, S11, S23, S25, and S31). For example, ACT- and ACC-based interventions disrupted fixation on negative cognitions through introspective observation of thoughts (e.g., S5, S6, and S20). This process was also used to address sensitivity to peer evaluation and psychological resistance to developmental bodily changes (S27).

Second, bodily anchoring and interoceptive awareness constituted another frequently identified therapeutic component, mainly targeting excessive physiological arousal, unnecessary muscular tension, and the resulting attentional narrowing. Under stage pressure, physiological reactions such as palpitations, rapid breathing, trembling, and bodily stiffness are often further interpreted as signs that one is losing control or about to perform poorly, thereby intensifying the anxiety cycle. Practices such as yoga postures, body scan, breath regulation, and grounding techniques may help individuals re-anchor attention to breathing, posture, and bodily sensations and may reduce threat-based interpretations of physiological arousal (e.g., S1, S3, S7, S24, and S26). In some studies, such mind-body training was also associated with greater bodily self-efficacy, relaxation, and a stronger sense of stability during performance (e.g., S8, S13, S17, S18, S19, and S22). In addition, neuroimaging evidence suggested that brief mindfulness training may alter neural connectivity involved in emotional processing, thereby alleviating MPA (S15).

Third, acceptance and values clarification specifically targeted experiential avoidance, behavioral procrastination, and psychological rigidity in anxiety-provoking situations. When anxiety arises, individuals often instinctively avoid performance in order to obtain temporary relief. However, this short-term reduction in distress may reinforce anxiety over time. ACT-based studies consistently suggested that training in acceptance, values orientation, and committed action does not require anxiety to disappear first; rather, it encourages individuals to continue moving toward personally meaningful performance goals even in the presence of anxiety. This mechanism was reflected in increased psychological flexibility, reduced avoidance, and greater capacity to tolerate performance-related distress (e.g., S4, S14, and S20). At the same time, meditation practices such as Zen meditation and silent illumination encouraged nonjudgmental acceptance of anxiety and, in some cases, even transformed it into a source of focused motivational energy (S23). Mindfulness courses integrated with vocal training also appeared to strengthen performers’ capacity to tolerate unexpected mistakes, such as voice cracks (S17 and S19).

Finally, self-compassion and positive resource building emerged as another important therapeutic component in many studies. This component mainly targeted maladaptive perfectionism, disturbed inner self-relations, low psychological safety, and limited emotional buffering resources. For many performers, anxiety arises not only from immediate stage demands but also from deeper self-evaluative patterns, such as chronic self-criticism, low tolerance for imperfection, and defining self-worth in terms of performance. Compassion-based interventions and the cultivation of trait mindfulness may buffer perfectionistic concerns and vulnerability to anxiety by strengthening self-soothing, self-acceptance, and a nonpunitive relationship with the self (e.g., S2, S12, S18, and S31). At the same time, multicomponent programs such as CRAFT, through building positive psychological resources such as gratitude and savoring, significantly enhanced musicians’ emotion regulation capacities during major crises such as pandemic-related lockdowns and transformed self-driven motivation into more enduring psychological resilience (e.g., S1, S9, S16, and S32). These interventions therefore targeted not only anxiety symptoms themselves but also the broader cultivation of inner safety, resilience, wellbeing, and a less self-critical way of relating to oneself, thereby indirectly buffering performance anxiety.

## 5 Discussion

This review adopted a dual-track framework that combined three-level meta-analysis with qualitative synthesis to estimate the effects of MABIs in music performance settings and to examine potential boundary conditions. Based on 32 independent studies, this review quantitatively assessed the effects of MABIs on reducing performers’ anxiety-related outcomes and improving positive psychological functioning, and, to some extent, explored possible mechanism-related commonalities across different intervention formats.

### 5.1 Characteristics of the current evidence base and future directions

The included literature indicates a marked upward trend in mindfulness- and acceptance-based intervention research with musicians; however, the current empirical base remains constrained by geographical and methodological characteristics. Geographically, existing studies are mainly concentrated in Western high-income countries, particularly the United States and the United Kingdom, where medical, psychological, and educational resources are relatively well developed. This distribution suggests the need for further validation in more diverse cultural and educational contexts. Methodologically, the current evidence base is overall of moderate strength, with most studies relying on nonrandomized controlled designs and one-group pretest-posttest designs. Although the leave-one-out analyses and publication-bias diagnostics indicated, to some extent, that the main effect estimates were not clearly driven by a single study or by evident funnel-plot asymmetry, these diagnostic results do not remove the study-design limitations identified in the JBI appraisal. Therefore, the findings should still be interpreted with caution.

These limitations in sampling and design reflect the real-world ecological constraints of conservatory and higher music education contexts. Because higher music education has long depended on one-to-one and small-group instructional structures (Bjøntegaard, 2015), researchers often face restricted participant pools and substantial difficulty implementing strict random allocation when recruiting and delivering interventions. Future research therefore needs to move beyond compromises associated with convenience sampling and conduct rigorously randomized, multicenter trials with active control conditions in order to strengthen the causal evidence chain.

### 5.2 The potential dual benefits of MABIs in music performance contexts

At the level of the main effects, the quantitative findings were consistent with H1 and H2, indicating that MABIs were associated with reduced anxiety-related outcomes and improved positive psychological functioning among music performers, while also suggesting that their effects may be subject to certain boundaries.

Regarding H1, MABIs produced a moderate-to-large reduction in overall anxiety outcomes. Exploratory analyses revealed significant effects for both outcome families, though the effect was more pronounced for MPA-specific anxiety. This pattern aligns with findings on other context-related anxieties (e.g., test or sport anxiety; Niering et al., 2023; Wang et al., 2024) and appears numerically larger than estimates for generalized anxiety (Khoury et al., 2013). This difference in magnitude may stem from MPA’s link to identifiable performance stressors, which makes attentional and acceptance-based skills more closely aligned with the measured anxiety episode. Specifically, under pressure, mindfulness-induced attentional re-anchoring can redirect focus from threat cues back to the musical task (Bishop et al., 2004), mitigating threat-focused processing (Eysenck et al., 2007) and automatic catastrophic responses (Osborne and Franklin, 2002). However, given the smaller and more heterogeneous sample of the broader anxiety family, this comparative finding warrants cautious interpretation.

On the other hand, with respect to H2, MABIs showed a small-to-moderate but statistically significant improvement within the broad adaptive-functioning umbrella domain, which is broadly consistent with the wider empirical literature on mindfulness-based interventions (Eberth and Sedlmeier, 2012; Stenhoff et al., 2020). However, this effect was clearly smaller than the reduction observed for anxiety-related outcomes. The exploratory analysis showed significant improvements in both proximal adaptive processes and distal performance and wellbeing resources, with relatively comparable gains across the two outcome families. Although these two subdomains are conceptually distinct, the current data do not support a clear differential intervention effect of MABIs between them. This relatively modest overall gain is partly consistent with Keyes’s (2002) dual-continua model, which suggests that the reduction of distress does not automatically imply the emergence of psychological flourishing. Future intervention studies should further examine whether explicitly integrating values-based action, self-compassion training, or performance-oriented resource building may help support more robust and sustained positive psychological adaptation. Related longitudinal evidence on loving-kindness compassion, mindfulness, and psychological wellbeing also suggests that these resource-oriented processes may be better understood through designs that track change over time (Ly et al., 2026).

### 5.3 Moderator findings and interpretive boundaries

#### 5.3.1 Intervention characteristics and mechanism-related components of MABIs

The present study found that intervention type did not significantly moderate the main effect. This result did not provide statistical support for H3a. Nevertheless, it remains compatible with the possibility that different MABI formats may share therapeutic components, although it should not be interpreted as evidence of intervention equivalence. This nonsignificant result may reflect limited statistical power, broad intervention categories, or residual heterogeneity. Therefore, the current findings are better understood as suggesting the possible presence of shared components rather than as confirming cross-format convergence.

Although the included interventions differed in their surface procedures—for example, yoga-based approaches emphasized the body, whereas ACT-based approaches often emphasized metaphor and experiential exercises—they commonly included four recurring therapeutic components: cognitive defusion and decentering, bodily anchoring and interoceptive awareness, acceptance and values-based action, and self-compassion and positive resource building. These components should not be reduced to broad relaxation techniques; rather, they may be conceptually linked with multidimensional processes associated with MPA, namely cognitive fusion, heightened physiological arousal, experiential avoidance, and maladaptive perfectionism.

This component-level synthesis is broadly consistent with observations from recent reviews (Bakhtiari et al., 2025; Kinney et al., 2025). More specifically, the cognitive defusion pathway is consistent with recent evidence on the role of decentering (Guo et al., 2024). In addition, incorporating self-compassion and values-based action into the set of shared components may provide a possible theoretical supplement for understanding the transition from alleviating negative distress to supporting psychological flourishing (Keyes, 2002). However, these components were mainly derived from intervention descriptions, qualitative materials, and theoretical explanations. They should therefore be interpreted as inferred mechanism-related components rather than causal mechanisms directly verified through mediation analysis or experimental manipulation. Thus, despite their conceptual overlap, whether specific formats can produce equivalent intervention benefits still requires direct comparison in future trials with sufficient statistical power.

#### 5.3.2 Intervention dose characteristics and dose–response patterns

The results further showed that intervention dose did not significantly moderate the overall effect size. This result did not provide statistical support for H3b, although it does not rule out the possibility of nonlinear or more fine-grained dose–response patterns. From a theoretical perspective, because MABIs emphasize facilitating a qualitative shift in individuals’ relationship with internal experiences, their effect trajectory may not follow a simple linear pattern. This preliminary finding invites reflection on the conventional assumption that “longer practice necessarily produces better outcomes”: once individuals have acquired an initial capacity for decentering and experienced a substantive shift in internal perspective, simply extending the number of intervention weeks may not necessarily lead to a linear increase in effect size. At the practical level, this suggests that appropriately dosed modular interventions may still have practical value within the compact curriculum structure of music conservatories.

However, this non-significant result must be interpreted with considerable caution. As Kinney et al. (2025) noted, the studies currently available in this field are generally characterized by small sample sizes, which substantially limits the statistical power of meta-regression analyses to detect small moderator effects. In addition, broad dose categorizations can easily obscure heterogeneity introduced by micro-ecological factors, such as baseline anxiety level, instructor expertise, and adherence to between-session practice. Therefore, this result only indicates that, within the current limited and heterogeneous evidence base, no stable linear moderating effect of dose on intervention effects was observed. It is exploratory and hypothesis-generating in nature, and should not be interpreted as evidence that dose effects are absent or that different dose levels produce equivalent effects.

In addition, regarding the temporal maintenance, limited longitudinal evidence tentatively suggests that reductions in MPA can be sustained for several months to a year post-intervention (e.g., Clarke et al., 2020; Fraser, 2025; Stern et al., 2012). If a qualitative shift across a possible efficacy threshold occurs, it could theoretically help explain why some benefits persist after formal training concludes. Nevertheless, current follow-up observations alone cannot establish such trait-level psychological restructuring. Therefore, future large-scale longitudinal research should incorporate higher-resolution metrics—such as single-session duration, exact homework volume, adherence, and intervention quality. These refined designs would help identify the minimum effective dose and potential non-linear inflection points more rigorously, and examine how baseline characteristics (e.g., initial anxiety severity and prior intervention experience) may moderate long-term benefits, ultimately informing more tailored, evidence-based guidelines for musicians.

### 5.4 Limitations

Several limitations should be taken into account when interpreting and applying the findings of this review. First, the literature search was restricted to English-language publications, which may introduce potential language bias and may have excluded relevant localized intervention evidence reported in other languages. Second, because of the constraints of authentic educational ecologies, the included empirical studies were generally characterized by moderate methodological limitations. Most quantitative studies lacked rigorous random assignment or active control groups, and small sample sizes were common, thereby limiting the strength of causal inference regarding intervention effects. Although the leave-one-out analyses suggested that the main pooled estimates were not driven by any single study, and the publication-bias diagnostics did not show statistically significant Egger tests or clear funnel-plot asymmetry, this does not remove concerns related to nonrandomized designs, uncontrolled pre-post comparisons, or incomplete follow-up reporting. Third, there was substantial heterogeneity across studies in both measurement selection and outcome definition. For example, different dimensions of MPA and diverse indicators of positive psychological functioning were used across studies, which may have reduced the comparability of pooled effect-size estimation. Although this review prospectively employed three-level meta-analysis and robust variance estimation (RVE) to address the nesting and dependence of effect sizes, the quality of reporting in the primary literature limited the availability of objective fine-grained covariates for meta-regression, such as precise home-practice duration and prior intervention experience. As a result, the substantial heterogeneity observed in the models could not be fully explained.

Taken together, future research would benefit from broader language coverage, improved sampling and comparison conditions where feasible, and higher-resolution monitoring of dynamic covariates. These steps would help strengthen the evidence base while acknowledging the practical constraints of authentic music education and performance settings.

## 6 Future research

Given the characteristics and limitations of the current literature, future empirical studies and systematic reviews should advance along at least three directions.

First, existing research is heavily concentrated on university music students in Western developed countries, resulting in relatively homogeneous samples. Future reviews and primary studies should extend attention to both ends of the career trajectory: downward to children and adolescent music learners, with a focus on the early development of psychological coping resources, and upward to professional orchestra members or freelance musicians working under sustained pressure, with a focus on the role of MABIs in addressing longer-term stress, burnout-related symptoms, and identity-related distress. This contextual expansion aligns with recent research consensus: wellbeing is not merely a symptom-oriented outcome, but a lifestyle embedded within daily sustainable health practices (Nguyen and Tran, 2025a).

Second, although this review coded four recurring mechanism-related components, the current evidence still relies heavily on self-report measures and qualitative interviews. With the exception of a few emerging studies that have incorporated physiological indicators (e.g., Boileau et al., 2025; Hague and Sandage, 2016), this field still lacks sufficient convergent evidence from objective physiological or neurophysiological indicators. Future research therefore would benefit from multimodal assessment by integrating neurophysiological indicators such as heart rate variability (HRV) and electroencephalography (EEG). This would help clarify how intervention-related processes unfold over time and whether physiological arousal is associated with changes in attentional control and anxiety symptoms. Such evidence would be particularly valuable for developing more differentiated intervention recommendations for individuals with severe MPA.

Finally, most current studies focus on immediate post-intervention outcomes. Because long-term follow-up and longitudinal research remain scarce, it is still difficult to establish the long-term stability of intervention effects with sufficient confidence. Moreover, future longitudinal designs should aim to disentangle the potential sources of long-term benefit. More specifically, they should assess whether sustained change is related to enduring reductions in anxiety, continued mindfulness practice after the intervention, or nonspecific short-term factors.

In sum, future studies should include more diverse participant groups, multimodal assessment, and longer-term follow-up. These steps would help clarify the conditions under which MABIs are associated with sustained psychological change and how they may be adapted for musicians at different developmental stages.

## Data Availability

The original contributions presented in this study are included in the article/Supplementary material, further inquiries can be directed to the corresponding author.
