# Supplementary material for: Mindfulness- and acceptance-based interventions for music performance anxiety: a three-level meta-analysis of therapeutic effects, mechanisms, and boundary conditions
Source: Front Psychol. 2026 Jun 11;17:1851221. doi: 10.3389/fpsyg.2026.1851221 (PMC13293812; doi:10.3389/fpsyg.2026.1851221)
Supplement: Supplementary file 1 [file Table_1.docx]

Supplementary Material

**Table 1. Characteristics of included studies**

| No | Title | Author, Year | Study Design | Country | Duration | Age Group | Intervention Category |
| --- | --- | --- | --- | --- | --- | --- | --- |
| 1 | A Feasibility Study of a Program Integrating Mindfulness, Yoga, Positive Psychology, and Emotional Intelligence in Tertiary-Level Student Musicians | Bartos et al., 2022 | Single-arm pre-post | Spain | 25 weeks (50 min/week) | 19-26 | Multicomponent & Integrated Interventions |
| 2 | A preliminary investigation into self-compassion and compassion-based intervention for mental health in the performing arts. | Walton et al., 2025 | Mixed methods (Cross-sectional + Single-arm pre-post) | Australia | 3 weeks (online + daily) | Adults ≥18 | Pure Mindfulness & Meditation |
| 3 | A yoga intervention for music performance anxiety in conservatory students | Stern et al., 2012 | Single-arm pre-post | USA | 9 weeks (2x60 min/week + daily) | 18-29 | Yoga-based Interventions |
| 4 | Acceptance and commitment coaching for music performance anxiety: Piloting a 6-week group course with undergraduate dance and musical theatre students. | Mahony et al., 2022 | Mixed methods (Single-arm pre-post) | UK | 6 weeks (1 session/week) | 18-20 | ACT/ACC |
| 5 | Acceptance and commitment therapy for the treatment of music performance anxiety: A pilot study with student vocalists. | Juncos et al., 2017 | Single-arm pre-post | USA | 12 sessions | 19-31 | ACT/ACC |
| 6 | Acceptance and commitment therapy for the treatment of music performance anxiety: A single subject design with a university student. | Juncos & Markman, 2016 | Single-subject design | USA | 10 sessions | ~19-20 | ACT/ACC |
| 7 | An explanatory sequential pilot inquiry on music therapy and performance anxiety in university music education majors. | Clements-Cortés et al., 2024 | Mixed methods (Single-arm pre-post) | Canada | 6 weeks (60 min/week) | 21-33 | Multicomponent & Integrated Interventions |
| 8 | An integrative intervention program for college musicians and kinematics in cello playing | Lee et al., 2019 | Single-arm pre-post | USA | 15-16 weeks (2 hours/week) | Young adults | Multicomponent & Integrated Interventions |
| 9 | Developing resilience during the COVID-19 pandemic: Yoga and mindfulness for the well-being of student musicians in Spain. | Bartos et al., 2021 | CCT | Spain | 1 academic year (1 hour/week) | 18-39 | Multicomponent & Integrated Interventions |
| 10 | Effects of a yoga lifestyle intervention on performance-related characteristics of musicians: a preliminary study. | Khalsa et al., 2006 | CCT | USA | 8 weeks (weekly + daily opt.) | 21-30 | Yoga-based Interventions |
| 11 | Effects of Meditation on Music Performance Anxiety | Chang et al., 2003 | RCT | USA | 8 weeks (1.25 hr/week + 20 min/day) | 18-41 | Pure Mindfulness & Meditation |
| 12 | Effects of online choir or mindfulness interventions on auditory perception and well-being in middle-and older-aged adults during the COVID-19 pandemic: A randomized controlled trial. | Herschel et al., 2022 | RCT | USA | 10 weeks (1 hr/week + homework) | 50-65 | Pure Mindfulness & Meditation |
| 13 | Evaluating the effects of stress reduction techniques and Fitzmaurice Voicework® on physiologic markers and mental states related to performance anxiety in student actors. | Hague & Sandage, 2016 | CCT | USA | 2 semesters | 18-22 | Multicomponent & Integrated Interventions |
| 14 | Examining a group acceptance and commitment therapy intervention for music performance anxiety in student vocalists. | Clarke et al., 2020 | Single-arm pre-post | Australia | 6 weeks (2 hours/week) | 19-61 (M=20.3) | ACT/ACC |
| 15 | Examining how brief mindfulness training influences communication within the brain of musicians with music performance anxiety: A resting state fMRI study | Boileau et al., 2025 | CCT | Canada | 2 weeks | Adults | Pure Mindfulness & Meditation |
| 16 | Examining the CRAFT program’s impact on student musicians’ well-being compared to controls | Bartos et al., 2025 | CCT | Spain | 7 months (60-90 min/week) | Young adults | Multicomponent & Integrated Interventions |
| 17 | Integration of mindfulness practices in vocal training: Enhancing performance and well-being | Wang, 2025 | CCT | China | 1 semester | Young adults | Multicomponent & Integrated Interventions |
| 18 | Mindfulness for musicians: A mixed methods study investigating the effects of 8-week mindfulness courses on music students at a leading conservatoire | Czajkowski et al., 2022 | Mixed methods (Single-arm pre-post) | UK | 8 weeks | Young adults | Pure Mindfulness & Meditation |
| 19 | Mindfulness for Singers: A Mixed Methods Replication Study | Czajkowski et al., 2021 | Mixed methods (RCT) | UK | 8 weeks | Young adults | Pure Mindfulness & Meditation |
| 20 | Piloting a New Model for Treating Music Performance Anxiety: Training a Singing Teacher to Use Acceptance and Commitment Coaching With a Student | Shaw et al., 2020 | Single-subject design | UK | 6 sessions (60 min/session) | Young adults | ACT/ACC |
| 21 | Mindfulness music training as a buffer against music performance anxiety: Emotional regulation and technical precision in conservatory students. | Li et al., 2026 | RCT | China | 8 weeks (2x60 min/week + daily) | 18-23 (M=20.4) | Multicomponent & Integrated Interventions |
| 22 | Psychological skills and mindfulness training effects on the psychological wellbeing of undergraduate music students: An exploratory study. | Steyn et al., 2016 | CCT | South Africa | 7 weeks | Young adults | Multicomponent & Integrated Interventions |
| 23 | Silent illumination: a study on Chan (Zen) meditation, anxiety, and musical performance quality | Lin et al., 2008 | RCT | USA | 8 weeks (1 session/week + 20 min/day) | 18-41 (M=25.1) | Pure Mindfulness & Meditation |
| 24 | Yoga Ameliorates Performance Anxiety and Mood Disturbance in Young Professional Musicians | Khalsa et al., 2009 | CCT | USA | 8 weeks | Young adults (M=24.5) | Yoga-based Interventions |
| 25 | Yoga Enhances Positive Psychological States in Young Adult Musicians | Butzer et al., 2016 | CCT | USA | 8 weeks | Young adults (M~24) | Yoga-based Interventions |
| 26 | Yoga Reduces Performance Anxiety in Adolescent Musicians | Khalsa et al., 2013 | CCT | USA | 6 weeks (3x60 min/week) | Adolescents (M~16.4) | Yoga-based Interventions |
| 27 | Acceptance and commitment coaching for music performance anxiety in adolescent singers | Paul et al., 2024 | Qualitative (Interview) | UK | 6 sessions (45-60 min) over 3-4 mo | 13-17 | ACT/ACC |
| 28 | Meditating musicians: investigating the experience of music students and professional musicians in a brief mindfulness course to address music performance anxiety | Paese & Schiavio, 2025 | Qualitative (case study) | UK | 21-day pilot + 4 weeks (1 session/week) | 19-54 | Pure Mindfulness & Meditation |
| 29 | Meditation as a tool to counteract music performance anxiety from the experts' perspective | Paese & Egermann, 2024 | Qualitative (Interview) | UK | N/A | Adults | Pure Mindfulness & Meditation |
| 30 | Racial Imposter Syndrome and Music Performance Anxiety: A Case Study | Fraser, 2025 | Qualitative (case study) | Australia | 12 sessions (1 hour) over 2 years | 30s | ACT/ACC |
| 31 | Relationships Among Meditation, Perfectionism, Mindfulness, and Performance Anxiety Among Collegiate Music Students | Diaz, 2018 | Cross-sectional | USA | N/A | Young adults | Pure Mindfulness & Meditation |
| 32 | The CRAFT Program: Mindfulness and Yoga for Enhancing the Well-Being and Academic Experience of Higher Education Student Musicians | Bartos et al., 2024 | Mixed methods (Cross-sectional qualitative) | Spain | 1 academic year (1 hr/week) | 20-29 | Multicomponent & Integrated Interventions |

**Table 2. Moderator Analyses of the Effects of MABIs on Context-Specific State Anxiety**

| Moderator | Category | *k* studies | *k* effects | *g* | SE | 95% CI | *p* | Interaction test |
| --- | --- | --- | --- | --- | --- | --- | --- | --- |
| **Overall modeled sample** | — | **18** | **68** | **0.732** | 0.115 | [0.506, 0.958] | < .001 |  |
| **Study design** | RCT | 3 | 3 | 0.677 | 0.311 | [0.067, 1.287] | .030 | QM(2) = 0.108, *p* = .947 |
|  | CCT | 8 | 45 | 0.772 | 0.173 | [0.434, 1.110] | < .001 |  |
|  | Single-group pre-post | 7 | 20 | 0.701 | 0.202 | [0.306, 1.096] | < .001 |  |
| **Intervention type** | Pure Mindfulness & Meditation | 3 | 4 | 0.622 | 0.324 | [-0.014, 1.258] | .055 | QM(3) = 0.988, *p* = .804 |
|  | ACT/ACC | 3 | 7 | 0.961 | 0.338 | [0.298, 1.624] | .005 |  |
|  | Yoga-based Interventions | 4 | 23 | 0.588 | 0.244 | [0.110, 1.067] | .016 |  |
|  | Multicomponent & Integrated Interventions | 8 | 34 | 0.778 | 0.176 | [0.433, 1.123] | < .001 |  |
| **Intervention dose** | Low-dose | 5 | 13 | 0.760 | 0.239 | [0.292, 1.228] | .001 | QM(2) = 0.139, *p* = .933 |
|  | Standard-dose | 5 | 13 | 0.655 | 0.240 | [0.184, 1.126] | .006 |  |
|  | High-dose | 8 | 42 | 0.758 | 0.173 | [0.419, 1.097] | < .001 |  |
